# The genome sequence of the Forest Cuckoo Bee, *Bombus sylvestris* (Lepeletier, 1832)

**DOI:** 10.12688/wellcomeopenres.18986.1

**Published:** 2023-02-15

**Authors:** Liam M. Crowley, University of Oxford and Wytham Woods Genome Acquisition, Darwin Tree of Life Barcoding collective, Wellcome Sanger Institute Tree of Life programme, Wellcome Sanger Institute Scientific Operations: DNA Pipelines collective, Tree of Life Core Informatics collective, Darwin Tree of Life Consortium

**Affiliations:** 1 University of Oxford, Oxfordshire, UK

**Keywords:** Bombus sylvestris, Forest Cuckoo Bee, genome sequence, chromosomal, Hymenoptera

## Abstract

We present a genome assembly from an individual male *Bombus sylvestris* (the Forest Cuckoo Bee; Arthropoda; Insecta; Hymenoptera; Apidae). The genome sequence is 303 megabases in span. Most of the assembly is scaffolded into 24 chromosomal pseudomolecules. The mitochondrial genome has also been assembled and is 23.6 kilobases in length. Gene annotation of this assembly on Ensembl has identified 13,025 protein coding genes.

## Species taxonomy

Eukaryota; Metazoa; Ecdysozoa; Arthropoda; Hexapoda; Insecta; Pterygota; Neoptera; Endopterygota; Hymenoptera; Apocrita; Aculeata; Apoidea; Apidae; *Bombus*; *Psithyrus*; *Bombus sylvestris* (Lepeletier, 1832) (NCBI:txid30201).

## Background

The Forest Cuckoo Bee, *Bombus sylvestris*, is one of six ‘cuckoo bumblebee’ species in the UK. It is a social parasite of *Bombus pratorum*, and possibly also *B. jonellus* and *B. monticola*, usurping colonies of these species and using the workers to raise its own offspring. This species does not produce workers. Females search out and enter a host nest, cohabiting in the nest before dominating the host queen and preventing oviposition of host eggs ( Küpper & Schwammberger, 1995). The *B. sylvestris* female then lays her own eggs in the host nest, that are reared by the host workers. Cuckoo bumblebees were formerly placed in their own genus *Psithyrus*, which has subsequently dropped to the rank of subgenus ( Pedersen, 1996).

*Bombus sylvestris* is widespread throughout much of Europe and can be found across the same habitats as its host(s), although it is rarely common. It is a small species – around 15 mm in length – and is covered in black hairs with a band of yellow hairs across the pronotum and white hairs on the apical segments of the abdomen. The white hairs are variably buff/darker in some individuals. The apex of the abdomen is strongly curled under in the female. Males have a tuft of orange hairs on the very tip of the abdomen and may have some buff/yellow hairs on tergite one ( Edwards & Jenner, 2005). Males produce a distinctive ‘mousy’ scent that is detectable by humans, derived from cephalic secretions related to territory marking ( Kullenberg *et al.*, 1970).

The phenology of this species matches the host species, with overwintered females emerging from late march and new males and females produced from July to September. It is likely to be bivoltine in areas where the host is also bivoltine, although this remains unclear. A wide range of flowers are visited for nectar. Pollen is not collected, although females may feed on pollen to facilitate ovary development.

A complete genome sequence for *B. sylvestris* will facilitate studies into the evolution of social parasitism and reproductive systems, as well as conservation of pollinator species.

## Genome sequence report

**Figure 1.**
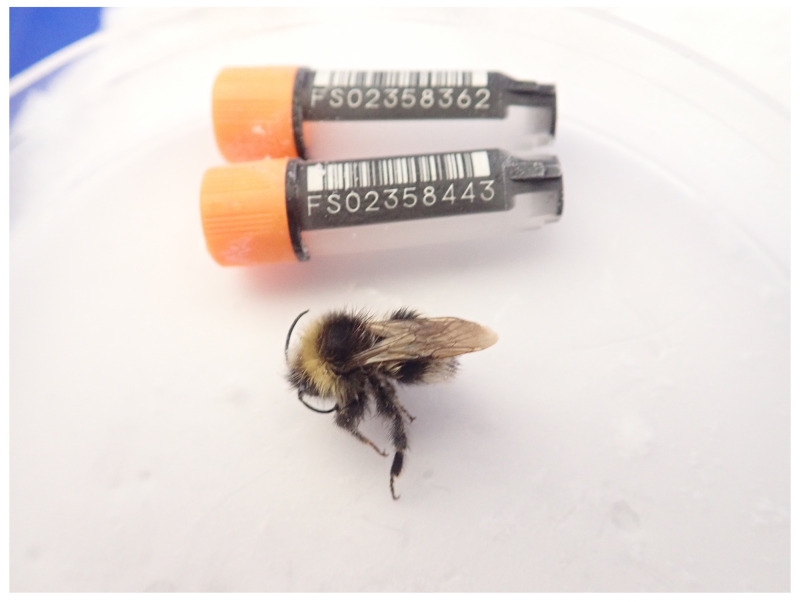
Photograph of the *Bombus sylvestris* (iyBomSyle1) specimen used for genome sequencing.

The genome was sequenced from one male *Bombus sylvestris* ( Figure 1) collected from Wytham Woods, Oxfordshire (latitude 51.78, longitude –1.34). A total of 79-fold coverage in Pacific Biosciences single-molecule HiFi long reads and 74-fold coverage in 10X Genomics read clouds were generated. Primary assembly contigs were scaffolded with chromosome conformation Hi-C data. Manual assembly curation corrected 48 missing joins or mis-joins, increasing the assembly length by 1.72%, reducing the scaffold number by 5.41%, and increasing the scaffold N50 by 80.9%.

**Figure 2.**
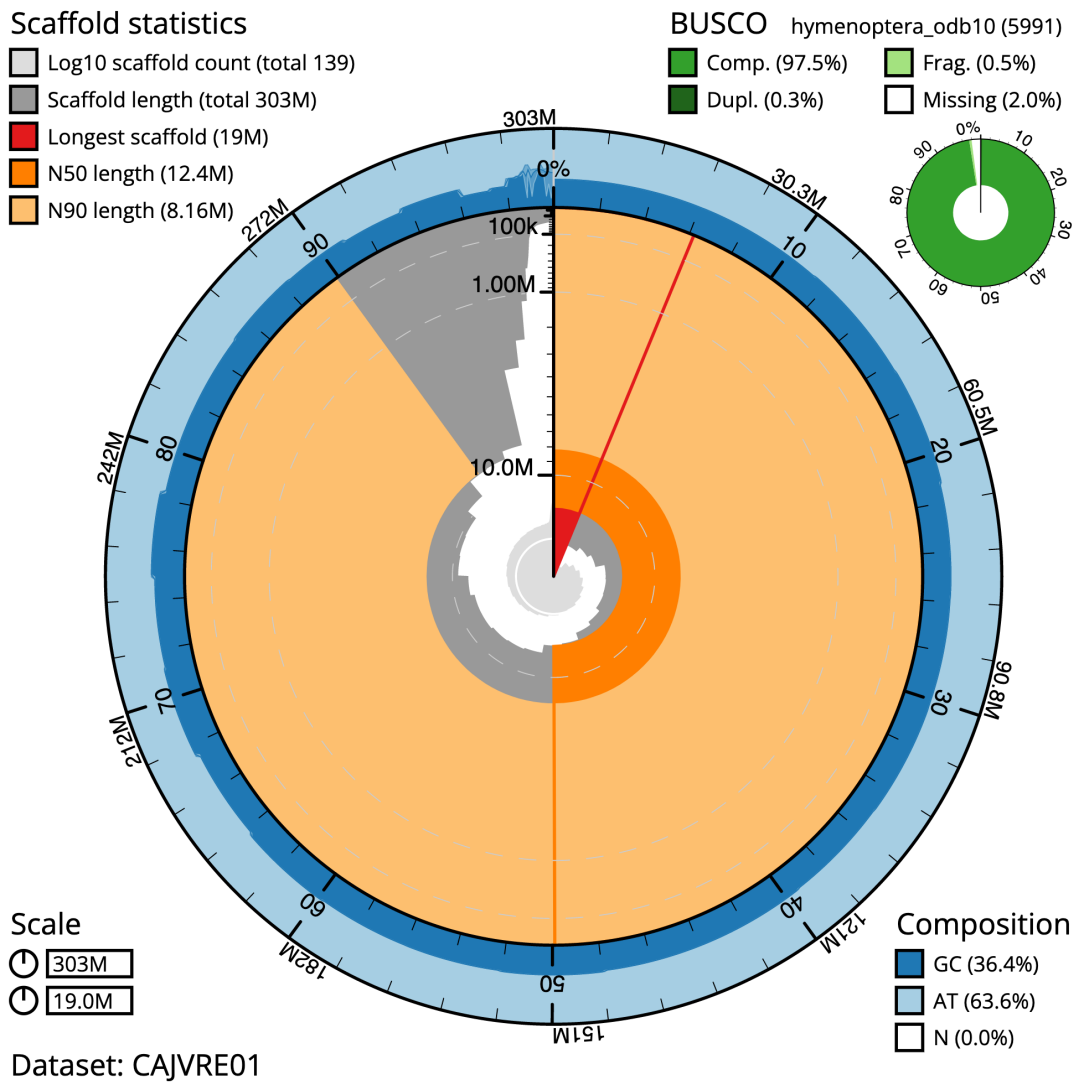
Genome assembly of *Bombus sylvestris*, iyBomSyle1.2: metrics. The BlobToolKit Snailplot shows N50 metrics and BUSCO gene completeness. The main plot is divided into 1,000 size-ordered bins around the circumference with each bin representing 0.1% of the 302,565,711 bp assembly. The distribution of scaffold lengths is shown in dark grey with the plot radius scaled to the longest scaffold present in the assembly (18,982,394 bp, shown in red). Orange and pale-orange arcs show the N50 and N90 scaffold lengths (12,445,291 and 8,163,122 bp), respectively. The pale grey spiral shows the cumulative scaffold count on a log scale with white scale lines showing successive orders of magnitude. The blue and pale-blue area around the outside of the plot shows the distribution of GC, AT and N percentages in the same bins as the inner plot. A summary of complete, fragmented, duplicated and missing BUSCO genes in the hymenoptera_odb10 set is shown in the top right. An interactive version of this figure is available at https://blobtoolkit.genomehubs.org/view/iyBomSyle1.1/dataset/CAJVRE01/snail.

The final assembly has a total length of 302.6 Mb in 140 sequence scaffolds with a scaffold N50 of 12.4 Mb ( Table 1). Most (97.15%) of the assembly sequence was assigned to 24 chromosomal-level scaffolds. Chromosome-scale scaffolds confirmed by the Hi-C data are named in order of size ( Figure 2– Figure 5; Table 2). The assembly has a BUSCO v5.3.2 ( Manni *et al.*, 2021) completeness of 97.5% (single 97.2%, duplicated 0.3%) using the hymenoptera_odb10 reference set ( *n* = 5,911).

**Table 1.** Genome data for *Bombus sylvestris*, iyBomSyle1.2.

Project accession data		
Assembly identifier	iyBomSyle1.2	
Species	*Bombus sylvestris*	
Specimen	iyBomSyle1	
NCBI taxonomy ID	30201	
BioProject	PRJEB45124	
BioSample ID	SAMEA7520657	
Isolate information	male; head and thorax (PacBio, 10X and Hi-C); abdomen (RNA-Seq)	
Assembly metrics *		*Benchmark*
Consensus quality (QV)	51	*≥ 50*
*k*-mer completeness	99.97%	*≥ 95%*
BUSCO **	C:97.5%[S:97.2%,D:0.3%], F:0.5%,M:2.0%,n:5,991	*C ≥ 95%*
Percentage of assembly mapped to chromosomes	97.15%	*≥ 95%*
Sex chromosomes	N/A	*localised homologous pairs*
Organelles	Mitochondrial genome assembled	*complete single alleles*
Raw data accessions		
PacificBiosciences SEQUEL II	ERR6412372	
10X Genomics Illumina	ERR6054792–ERR6054795	
Hi-C Illumina	ERR6054791	
PolyA RNA-Seq Illumina	ERR6286721	
Genome assembly		
Assembly accession	GCA_911622165.2	
Span (Mb)	302.6	
Number of contigs	193	
Contig N50 length (Mb)	5.9	
Number of scaffolds	140	
Scaffold N50 length (Mb)	12.4	
Longest scaffold (Mb)	19.0	
**Genome annotation**		
Number of protein-coding genes	13,025	
Average length of coding sequence (bp)	12,696.31	
Average number of exons per transcript	6.12	
Average number of introns per transcript	5.12	
Average intron size (bp)	1,644.29	

* Assembly metric benchmarks are adapted from column VGP-2020 of “Table 1: Proposed standards and metrics for defining genome assembly quality” from ( Rhie *et al.*, 2021). ** BUSCO scores based on the hymenoptera_odb10 BUSCO set using v5.3.2. C = complete [S = single copy, D = duplicated], F = fragmented, M = missing, n = number of orthologues in comparison. A full set of BUSCO scores is available at https://blobtoolkit.genomehubs.org/view/iyBomSyle1.1/dataset/CAJVRE01/busco.

**Figure 3.**
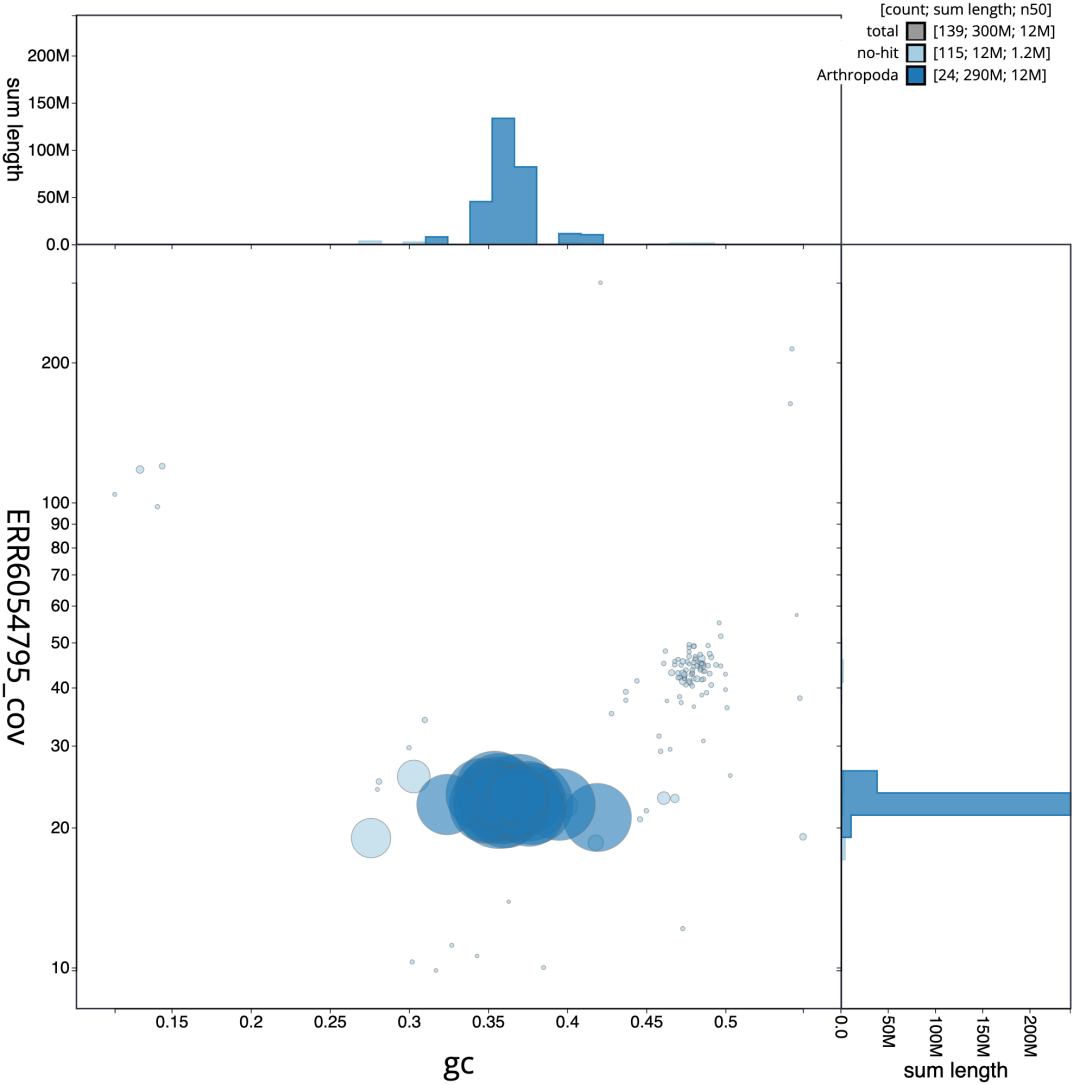
Genome assembly of *Bombus sylvestris*, iyBomSyle1.2: GC coverage. BlobToolKit GC-coverage plot. Scaffolds are coloured by phylum. Circles are sized in proportion to scaffold length. Histograms show the distribution of scaffold length sum along each axis. An interactive version of this figure is available at https://blobtoolkit.genomehubs.org/view/iyBomSyle1.1/dataset/CAJVRE01/blob.

**Figure 4.**
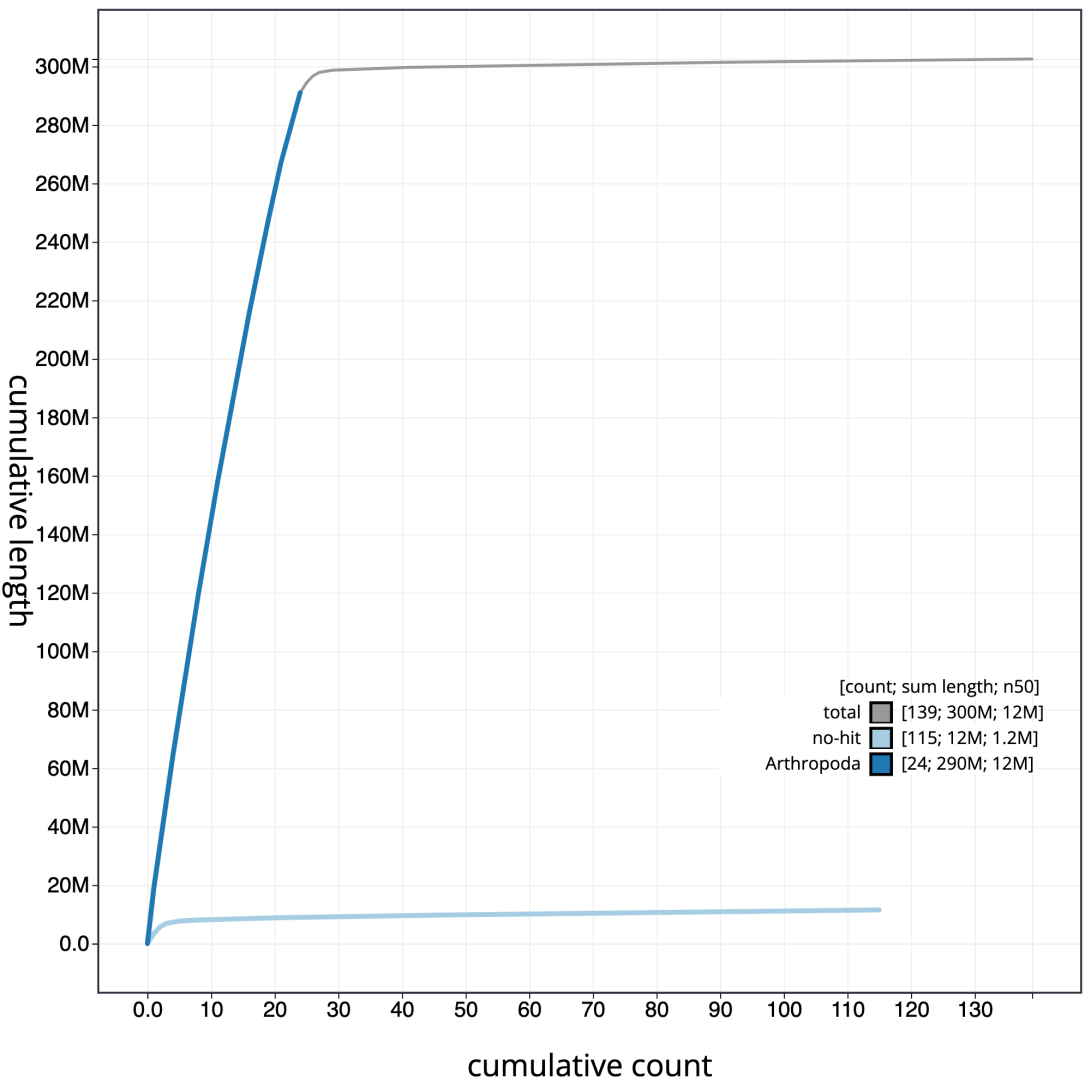
Genome assembly of *Bombus sylvestris*, iyBomSyle1.2: cumulative sequence. BlobToolKit cumulative sequence plot. The grey line shows cumulative length for all scaffolds. Coloured lines show cumulative lengths of scaffolds assigned to each phylum using the buscogenes taxrule. An interactive version of this figure is available at https://blobtoolkit.genomehubs.org/view/iyBomSyle1.1/dataset/CAJVRE01/cumulative.

**Figure 5.**
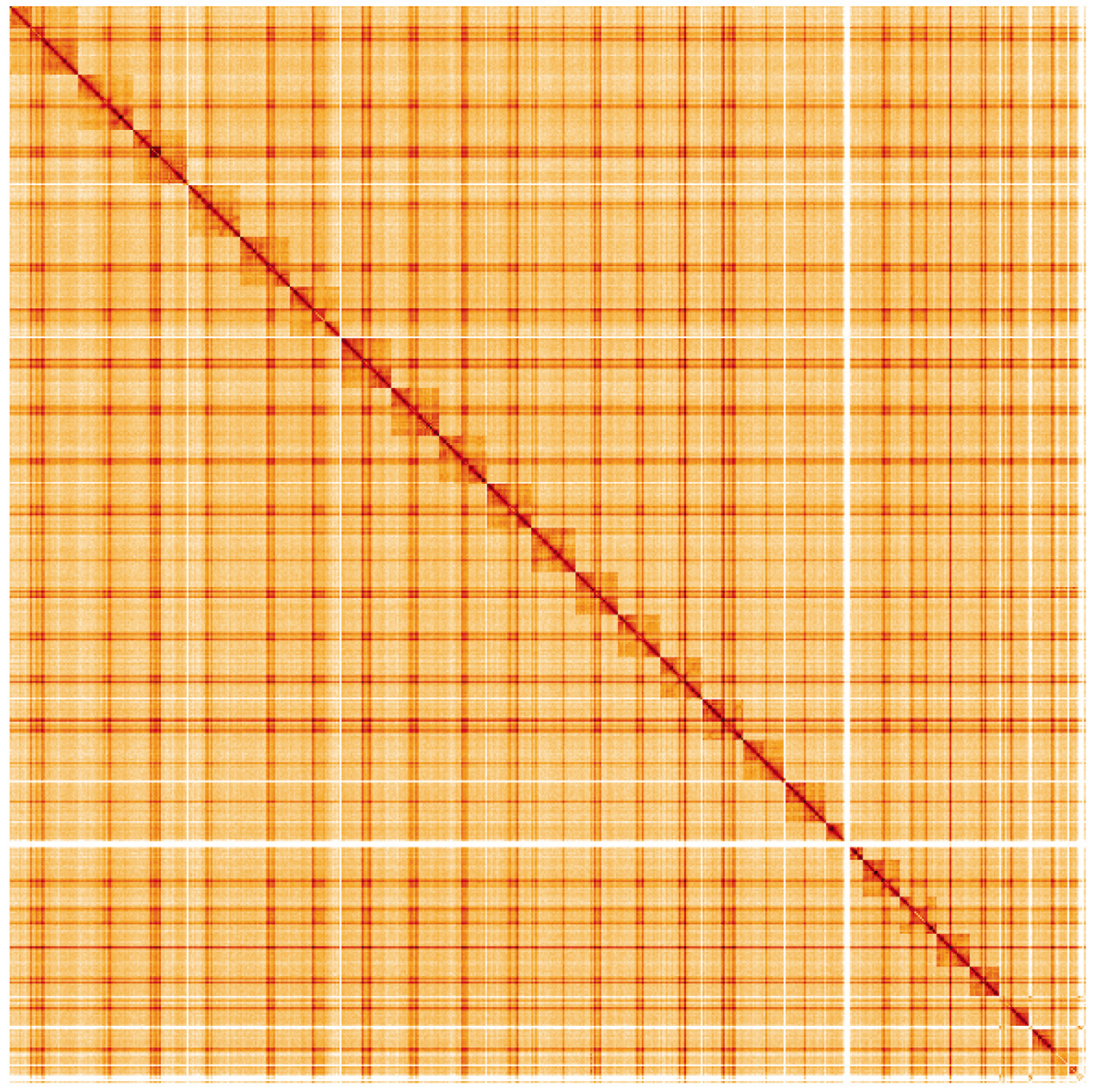
Genome assembly of *Bombus sylvestris*, iyBomSyle1.2: Hi-C contact map. Hi-C contact map of the iyBomSyle1.2 assembly, visualised using HiGlass. Chromosomes are shown in order of size from left to right and top to bottom. An interactive version of this figure may be viewed at https://genome-note-higlass.tol.sanger.ac.uk/l/?d=DBoJSZEqSRaXzTmtjCWZ4A.

**Table 2.** Chromosomal pseudomolecules in the genome assembly of *Bombus sylvestris*, iyBomSyle1.

INSDC accession	Chromosome	Size (Mb)	GC%
OU443141.1	1	18.98	35.7
OU443142.1	2	15.57	35.8
OU443143.1	3	15.11	36.9
OU443144.1	4	14.74	37.8
OU443145.1	5	13.92	35.7
OU443146.1	6	13.88	35.4
OU443147.1	7	13.83	35.7
OU443148.1	8	13.45	35
OU443158.1	9	10.35	41.9
OU443149.1	10	13.16	36.1
OU443150.1	11	12.57	36.1
OU443151.1	12	12.45	37.6
OU443152.1	13	11.79	36.6
OU443153.1	14	11.69	36.4
OU443154.1	15	11.62	37.5
OU443155.1	16	11.48	34.6
OU443156.1	17	11.47	39.5
OU443157.1	18	11.14	37.6
OU443159.1	19	10.29	35.2
OU443160.1	20	10.24	35
OU443161.1	21	9.47	36.7
OU443162.1	22	8.16	35.5
OU443163.1	23	8.11	32.3
OU443164.1	24	7.57	36.9
OX346368.1	MT	0.02	12
-	unplaced	11.52	36.2

## Genome annotation report

Annotation of the *B. sylvestris* GCA_911622165.1 assembly was generated using the Ensembl genome annotation pipeline ( Table 1; https://rapid.ensembl.org/Bombus_sylvestris_GCA_911622165.1/). The resulting annotation includes 13,025 protein coding genes with an average length of 12,696.31 and an average coding length of 1,413.16, and 5,798 non-protein coding genes. There is an average of 6.12 exons and 5.12 introns per canonical protein coding transcript, with an average intron length of 1,644.29. A total of 7,657 gene loci have more than one associated transcript.

## Methods

### Sample acquisition and nucleic acid extraction

A single male *Bombus sylvestris* (iyBomSyle1) was collected by netting in Wytham Woods, Oxfordshire (biological vice-county: Berkshire), UK (latitude 51.78, longitude –1.34) on 1 June 2020. The specimen was collected and identified by Liam Crowley (University of Oxford) and then snap-frozen on dry ice.

DNA was extracted at the Tree of Life laboratory, Wellcome Sanger Institute (WSI). The iyBomSyle1 sample was weighed and dissected on dry ice with tissue set aside for Hi-C sequencing. Head and thorax tissue was cryogenically disrupted to a fine powder using a Covaris cryoPREP Automated Dry Pulveriser, receiving multiple impacts. High molecular weight (HMW) DNA was extracted using the Qiagen MagAttract HMW DNA extraction kit. Low molecular weight DNA was removed from a 20 ng aliquot of extracted DNA using 0.8X AMpure XP purification kit prior to 10X Chromium sequencing; a minimum of 50 ng DNA was submitted for 10X sequencing. HMW DNA was sheared into an average fragment size of 12–20 kb in a Megaruptor 3 system with speed setting 30. Sheared DNA was purified by solid-phase reversible immobilisation using AMPure PB beads with a 1.8X ratio of beads to sample to remove the shorter fragments and concentrate the DNA sample. The concentration of the sheared and purified DNA was assessed using a Nanodrop spectrophotometer and Qubit Fluorometer and Qubit dsDNA High Sensitivity Assay kit. Fragment size distribution was evaluated by running the sample on the FemtoPulse system.

RNA was extracted from abdomen tissue of iyBomSyle1 in the Tree of Life Laboratory at the WSI using TRIzol, according to the manufacturer’s instructions. RNA was then eluted in 50 μl RNAse-free water and its concentration assessed using a Nanodrop spectrophotometer and Qubit Fluorometer using the Qubit RNA Broad-Range (BR) Assay kit. Analysis of the integrity of the RNA was done using Agilent RNA 6000 Pico Kit and Eukaryotic Total RNA assay.

### Sequencing

Pacific Biosciences HiFi circular consensus and 10X Genomics read cloud DNA sequencing libraries were constructed according to the manufacturers’ instructions. Poly(A) RNA-Seq libraries were constructed using the NEB Ultra II RNA Library Prep kit. DNA and RNA sequencing was performed by the Scientific Operations core at the WSI on Pacific Biosciences SEQUEL II (HiFi), Illumina HiSeq 4000 (RNA-Seq) and HiSeq X Ten (10X) instruments. Hi-C data were also generated from tissue of iyBomSyle1 using the Arima v2 kit and sequenced on the HiSeq X Ten instrument.

### Genome assembly

Assembly was carried out with Hifiasm ( Cheng *et al.*, 2021) and haplotypic duplication was identified and removed with purge_dups ( Guan *et al.*, 2020). One round of polishing was performed by aligning 10X Genomics read data to the assembly with Long Ranger ALIGN, calling variants with freebayes ( Garrison & Marth, 2012). The assembly was then scaffolded with Hi-C data ( Rao *et al.*, 2014) using SALSA2 ( Ghurye *et al.*, 2019). The assembly was checked for contamination and corrected using the gEVAL system ( Chow *et al.*, 2016) as described previously ( Howe *et al.*, 2021). Manual curation was performed using gEVAL, HiGlass ( Kerpedjiev *et al.*, 2018) and Pretext ( Harry, 2022). The mitochondrial genome was assembled using MitoHiFi ( Uliano-Silva *et al.*, 2022), which performed annotation using MitoFinder ( Allio *et al.*, 2020). The genome was analysed and BUSCO scores were generated within the BlobToolKit environment ( Challis *et al.*, 2020). Table 3 contains a list of all software tool versions used, where appropriate.

**Table 3.** Software tools and versions used.

Software tool	Version	Source
BlobToolKit	3.5.0	Challis *et al.*, 2020
freebayes	1.3.1-17- gaa2ace8	Garrison & Marth, 2012
gEVAL	N/A	Chow *et al.*, 2016
Hifiasm	0.12	Cheng *et al.*, 2021
HiGlass	1.11.6	Kerpedjiev *et al.*, 2018
Long Ranger ALIGN	2.2.2	https://support.10xgenomics.com/genome-exome/ software/pipelines/latest/advanced/other-pipelines
MitoHiFi	1	Uliano-Silva *et al.*, 2022
PretextView	0.2	Harry, 2022
purge_dups	1.2.3	Guan *et al.*, 2020
SALSA	2.2	Ghurye *et al.*, 2019

### Genome annotation

The Ensembl gene annotation system ( Aken *et al.*, 2016) was used to generate annotation for the *B. sylvestris* assembly (GCA_911622165.1). Annotation was created primarily through alignment of transcriptomic data to the genome, with gap filling via protein to-genome alignments of a select set of proteins from UniProt ( UniProt Consortium, 2019).

### Ethics and compliance issues

The materials that have contributed to this genome note have been supplied by a Darwin Tree of Life Partner. The submission of materials by a Darwin Tree of Life Partner is subject to the Darwin Tree of Life Project Sampling Code of Practice. By agreeing with and signing up to the Sampling Code of Practice, the Darwin Tree of Life Partner agrees they will meet the legal and ethical requirements and standards set out within this document in respect of all samples acquired for, and supplied to, the Darwin Tree of Life Project. All efforts are undertaken to minimise the suffering of animals. Each transfer of samples is further undertaken according to a Research Collaboration Agreement or Material Transfer Agreement entered into by the Darwin Tree of Life Partner, Genome Research Limited (operating as the Wellcome Sanger Institute), and in some circumstances other Darwin Tree of Life collaborators.

## Data Availability

European Nucleotide Archive: *Bombus sylvestris* (forest cuckoo bee). Accession number PRJEB45124; https://identifiers.org/ena.embl/PRJEB45124. ( Wellcome Sanger Institute, 2021) The genome sequence is released openly for reuse. The *Bombus sylvestris* genome sequencing initiative is part of the Darwin Tree of Life (DToL) project. All raw sequence data and the assembly have been deposited in INSDC databases. Raw data and assembly accession identifiers are reported in Table 1.

## Author information

Members of the University of Oxford and Wytham Woods Genome Acquisition Lab are listed here: https://doi.org/10.5281/zenodo.4789928.

Members of the Darwin Tree of Life Barcoding collective are listed here: https://doi.org/10.5281/zenodo.4893703.

Members of the Wellcome Sanger Institute Tree of Life programme are listed here: https://doi.org/10.5281/zenodo.4783585.

Members of Wellcome Sanger Institute Scientific Operations: DNA Pipelines collective are listed here: https://doi.org/10.5281/zenodo.4790455.

Members of the Tree of Life Core Informatics collective are listed here: https://doi.org/10.5281/zenodo.5013541.

Members of the Darwin Tree of Life Consortium are listed here: https://doi.org/10.5281/zenodo.4783558.
