# Supplementary material for: The genome sequence of the Forest Cuckoo Bee, Bombus sylvestris (Lepeletier, 1832)
Source: Wellcome Open Res. 2023 Feb 15;8:78. [Version 1] doi: 10.12688/wellcomeopenres.18986.1 (PMC10594053; doi:10.12688/wellcomeopenres.18986.1)
Supplement: Peer review history

# Reviewer report: Reviewer response for version 1

**DOI:** 10.21956/wellcomeopenres.21049.r68202

**Published:** 2023-10-23

**Authors:** Shaoying Wu

**Affiliations:** 1 Hainan University, Haikou, China

The introduction briefly describes the remarkable biology of the species. The genome was produced using an excellent method, the manuscript provides the details of the analysis, the data is excellent, and the resulting genome is of high quality. This paper describes the genome sequencing of a social parasitic bumblebee, *Bombus sylvestris*. It can provide a rich database for entomologists around the world, especially a specialist in bees.

Are sufficient details of methods and materials provided to allow replication by others?

Yes

Is the rationale for creating the dataset(s) clearly described?

Yes

Are the datasets clearly presented in a useable and accessible format?

Yes

Are the protocols appropriate and is the work technically sound?

Yes

Reviewer Expertise:

NA

I confirm that I have read this submission and believe that I have an appropriate level of expertise to confirm that it is of an acceptable scientific standard.

# Reviewer report: Reviewer response for version 1

**DOI:** 10.21956/wellcomeopenres.21049.r66441

**Published:** 2023-09-28

**Authors:** Peter K Dearden

**Affiliations:** 1 University of Otago, Dunedin, Aotearoa,, New Zealand

This manuscript describes the genome sequencing of *Bombus sylvestris*, a socially parasitic bumblebee species. The genome has been produced using an excellent methodology and the manuscript provides details of the analysis, which are standard, and have produced a high-quality genome.

 The introduction provides a brief description of the remarkable biology of this species. The figures are excellent, and the resulting genome is high quality and scores highly with BUSCO analysis. 

 My only comment would be the HiC plot, figure 5, is usual, and it would be nice (though not crucial) to know what is causing that.

Are sufficient details of methods and materials provided to allow replication by others?

Yes

Is the rationale for creating the dataset(s) clearly described?

Yes

Are the datasets clearly presented in a useable and accessible format?

Yes

Are the protocols appropriate and is the work technically sound?

Yes

Reviewer Expertise:

Insect genomics

I confirm that I have read this submission and believe that I have an appropriate level of expertise to confirm that it is of an acceptable scientific standard.
